# Current Status of Grain Drying Technology and Equipment Development: A Review

**DOI:** 10.3390/foods14142426

**Published:** 2025-07-09

**Authors:** Pengpeng Yu, Wenhui Zhu, Chaoping Shen, Yu Qiao, Wenya Zhang, Yansheng Zhu, Jun Gong, Jianrong Cai

**Affiliations:** 1 School of Agricultural Engineering, Jiangsu University, Zhenjiang 212013, China; 2 School of Food and Biological Engineering, Jiangsu University, Zhenjiang 212013, China; 15393060631@163.com (W.Z.); qiaoyuqiaoyu@126.com (Y.Q.); 3 School of Energy and Power Engineering, Jiangsu University, Zhenjiang 212013, China; shenchaoping0521@163.com; 4 College of Water Resources and Civil Engineering, China Agricultural University, Beijing 100083, China; zwy312501001@163.com; 5 Zoomlion Agriculture Machinery Co., Ltd., Wuhu 241000, China; zhuys_mark@163.com (Y.Z.); gongjun@zoomlion-hm.com (J.G.)

**Keywords:** grain drying, drying technology, drying equipment, energy efficiency, agricultural processing

## Abstract

Grain drying technology is a core process for ensuring food quality, extending storage life, and improving processing adaptability. With the continuous growth of global food demand and the increasing requirements for food quality and energy efficiency, traditional drying technologies face multiple challenges. This paper reviews six major grain drying technologies, comprising hot air drying, microwave drying, infrared drying, freeze drying, vacuum drying, and solar drying. It provides an in-depth discussion of the working principles, advantages, and limitations of each technology, and analyzes their performance in practical applications. In response to challenges such as high energy consumption, uneven drying, and quality loss during the drying process, the paper also explores the research progress of several hybrid drying systems, such as microwave–hot air drying combined systems and solar–infrared drying systems. Although these emerging technologies show significant potential in improving drying efficiency, energy saving, and maintaining food quality, their high costs, scalability, and process stability still limit large-scale applications. Therefore, future research should focus on reducing energy consumption, improving drying precision, and optimizing drying system integration, particularly by introducing intelligent control systems. This would maximize the preservation of food quality while improving the system’s economic efficiency and sustainability, promoting innovation in food production and processing technologies, and further advancing global food security and sustainable agricultural development.

## 1. Introduction

**Figure 1:**
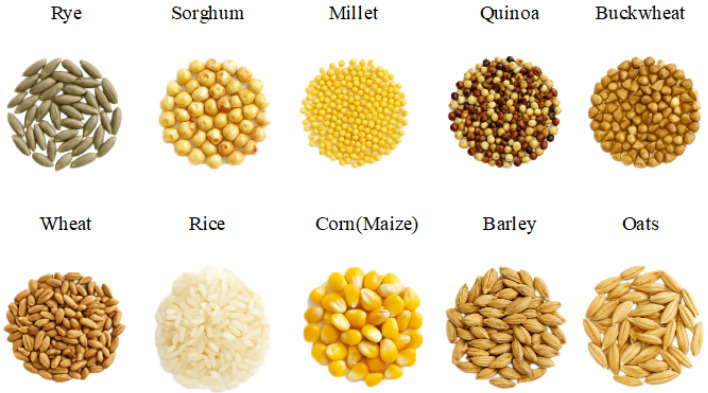
Various types of grains.

Grains are among the most important food crops globally, playing a crucial role in ensuring global food security and promoting sustainable agricultural development [1]. According to the Food and Agriculture Organization (FAO) of the United Nations, the annual production of grains accounts for nearly half of the total global food production, encompassing a variety of crops such as wheat, maize, rice, and legumes (Figure 1). With the continuous growth of the global population and the increasing demand for food, the yield and quality of grains have become focal points in global agricultural research. However, it is important to note that the yield of grains does not equate to their storage and distribution capabilities. At various stages of agricultural production, efficiently processing, preserving, and transporting grains has become a critical issue for ensuring the stability of the global food supply chain [2].

Grain drying is a critical process that directly affects its storage stability, processing adaptability, and the quality of the final product [3]. The drying process can significantly enhance the storage life and transportation efficiency of grains by effectively reducing their moisture content, inhibiting the growth of bacteria and mold, and preventing decay and spoilage [4]. This is particularly important in regions with high humidity, where timely drying can effectively prevent issues such as mold growth and pest infestations caused by excess moisture [5]. Additionally, a well-designed drying process can preserve the nutritional components of grains, maintaining their flavor and texture [6,7,8]. Therefore, research into and application of efficient, energy-saving, and low-loss drying technologies are of significant practical importance for promoting the sustainability of grain production and ensuring food security.

Despite the continuous development of modern grain drying technologies, numerous challenges still exist in practical applications, affecting their efficiency, energy consumption, and the quality of the final product [9]. One of the most common issues is low drying efficiency. Traditional drying methods often rely on convective hot air or heat conduction, which are relatively slow in drying speed, resulting in high energy consumption and low efficiency. This is particularly problematic in high-yield and large-scale production processes, where drying time and energy costs represent significant bottlenecks that cannot be overlooked [10,11].

Furthermore, energy consumption remains a long-standing challenge in the field of grain drying. Many traditional drying technologies, especially hot air drying, require a substantial amount of thermal energy to maintain the drying process, leading to enormous energy consumption. With the global energy transition and environmental policies gaining momentum, reducing energy waste and lowering carbon emissions during the drying process has become an urgent issue that needs to be addressed [12].

In addition, uneven drying, a common issue in grain drying processes, can also affect the quality of the final product. In traditional drying methods, the drying speed on the surface is usually faster, while the evaporation of moisture inside the grains occurs more slowly, causing a mismatch in drying rates between the surface and the interior. Over-drying can lead to increased hardness and a decline in taste, while under-drying may result in mold growth, negatively impacting the storage of grains [13].

Additionally, quality degradation during the drying process is a critical issue. Particularly in high-temperature drying, certain nutrients in grains, such as vitamins and minerals, may be lost due to excessive heat treatment, resulting in a decline in their nutritional value. Over-drying can also cause surface cracking or discoloration, adversely affecting the market value of the grains. Therefore, a key challenge in current drying technology research is how to efficiently dry grains while maximizing the retention of their nutritional and sensory qualities [14].

As grain drying technology continues to evolve, many emerging drying methods have been introduced and widely applied in practice. Thus, gaining a comprehensive understanding of the latest grain drying technologies, research advancements, and challenges is of significant importance for driving innovation in related fields. This paper aims to review the fundamental principles of grain drying, key technologies, current equipment applications, and development trends, with a particular focus on future research directions related to energy efficiency improvement, quality assurance, and intelligent control [15].

First, this paper will introduce the basic principles of grain drying, emphasizing the analysis of physical and chemical mechanisms such as heat transfer and moisture transfer during the drying process, as well as the variations in drying curves. Next, the paper will review and critically evaluate the current mainstream grain drying technologies, including traditional hot air drying [16], as well as modern methods, such as microwave drying, infrared drying, and freeze drying [17]. The advantages and disadvantages of each method will be analyzed in detail, along with the exploration of combined applications of different technologies. Following this, the application status and development trends of existing drying equipment will be discussed. Finally, the paper will look ahead to the future trends in grain drying technology, particularly focusing on cutting-edge technologies related to energy-saving, high-efficiency precision drying, green and environmentally friendly technologies, and intelligent drying systems.

Through this review, the paper aims to provide the academic and industrial communities with the latest research findings and practical experiences in grain drying technology, while also offering valuable references and guidance for future research.

## 2. Fundamental Principles of Grain Drying

Grain drying is a complex physical process involving both heat transfer and mass transfer [18]. Its primary objective is to remove moisture from the grains and control their moisture content to an optimal level, ensuring stability and safety during subsequent storage, transportation, and processing. During this process, heat and moisture transfer are intricately intertwined [19]. By precisely controlling the drying conditions, it is possible to achieve efficient drying while maximizing the retention of the grains’ nutritional content and sensory quality [20].

### 2.1. Heat and Mass Transfer Process

During the grain drying process, the heat and mass transfer processes are interrelated and consist of two main interacting mechanisms: heat transfer and mass transfer [21,22]. Heat transfer refers to the transfer of thermal energy from the drying medium to the surface of the grain, while mass transfer involves the movement of moisture from the interior of the grain to the surface, eventually evaporating into the surrounding environment [23]. These two processes are coupled, and any changes in one aspect can significantly affect the overall drying efficiency [24].

Heat Transfer Process: During drying, thermal energy enters the grain through convection, conduction, or radiation [25]. Convection is the most common method of heat transfer, where hot air comes into contact with the surface of the grain and conducts heat to the interior, causing moisture to evaporate. Heat conduction refers to the transfer of heat along the solid phase of the grain [26], and in microwave or infrared drying technologies, radiation also plays an important role. The rate of heat transfer varies across different drying methods, directly impacting both the drying rate and energy efficiency.

Convective heat transfer is typically described by Newton’s law of cooling [27]:

(1) $$Q=hA\left(T_{s}-T_{\infty }\right)$$

where: *Q* is the heat transfer rate (W). *h* is the convective heat transfer coefficient (W/m^2^·K)*. A* is the contact area (m^2^)*. T_s_* is the surface temperature (K). *T_∞_* is the fluid temperature (K).

Heat conduction is described by Fourier’s law [28]:

(2) $$Q=-kA\frac{\Delta T}{\Delta x}$$

where: *k* is the thermal conductivity of the material (W/m·K). Δ*T* is the temperature difference (K). Δ*x* is the distance over which heat is transferred (m).

Radiation heat transfer is described by the Stefan–Boltzmann law [29]:

(3) $$Q=\epsilon \sigma A\left(T_{s}^{4}-T_{\infty }^{4}\right)$$

where: *ϵ* is the emissivity of the material (dimensionless). *σ* is the Stefan-Boltzmann constant (5.67 × 10^−8^ W/m^2^·K^4^).

Mass Transfer Process: The migration and evaporation of moisture are key steps in grain drying. Moisture migration first depends on capillary action, which transports moisture from the interior of the grain to the surface; then, the moisture migrates to the surface and enters the vapor phase via diffusion and convective mechanisms. Evaporation occurs as gas molecules depart from the grain surface and diffuse into the surrounding air [30]. Moisture gradient, temperature gradient, and airflow rate are the main factors affecting the moisture transfer rate [23].

Moisture migration is described by Fick’s law of diffusion [31]:

(4) $$J=-D\frac{\partial C}{\partial x}$$

where: *J* is the moisture flux (kg/m^2^·s). *D* is the diffusion coefficient (m^2^/s). $\frac{\partial C}{\partial x}$ is the moisture concentration gradient (kg/m^3^·m).

Capillary action involves the migration of liquid water and is typically described by [32]:

(5) $$z=\frac{2\delta \cos\theta }{r\rho g}$$

where: *z* is the capillary pressure (Pa). δ is the surface tension of the liquid (N/m). *θ* is the contact angle (°). *r* is the capillary radius (m). *ρ* is the liquid density (kg/m^3^). *g* is the gravitational acceleration (m/s^2^).

The moisture evaporation rate is typically described by [33]:

(6) $$\dot{m}=v\cdot A\cdot \left(C_{s}-C_{\infty }\right)$$

where: *m* is the evaporation rate (kg/s). v is the mass transfer coefficient for evaporation (kg/m^2^·s·Pa). *A* is the evaporation area (m^2^). *C*_*s*_ and *C*_∞_ are the surface vapor concentration and environmental vapor concentration, respectively (Pa).

In the coupled heat and mass transfer process, heat transfer and moisture migration are closely related. Heat is transferred to the grain surface through conduction, convection, or radiation, while moisture migrates to the surface via capillary action and diffusion, and evaporates into the vapor phase driven by the temperature gradient. This process can be further described by heat and mass transfer coupling equations:

(7) $$\frac{\partial T}{\partial t}=\alpha_{T}\nabla^{2}T$$

(8) $$\frac{\partial C}{\partial t}=\alpha_{C}\nabla^{2}C$$

where: *T* is the temperature (K). *C* is the moisture concentration (kg/m^3^). *α*_*T*_ and *α*_*C*_ are the thermal and mass diffusion coefficients, respectively (m^2^/s). ∇^2^ is the Laplacian operator, representing spatial variation in the diffusion process.

### 2.2. Drying Curve and Stages of Drying

The grain drying process typically exhibits different stages, and the variation in drying rate over time can be described using a drying curve [34]. Based on changes in the drying rate, the drying process is commonly divided into three main stages [35]:

1. Constant rate drying period

During this stage, the evaporation rate of moisture from the grain surface remains relatively constant, primarily determined by the external heat input and the diffusion of water vapor. The surface moisture evaporates rapidly, and the drying rate in this phase is typically high. At this point, the internal moisture distribution remains stable, and there is a significant moisture gradient. The duration of the constant rate drying period depends on the initial moisture content of the grain and factors such as external temperature and humidity. The drying rate in this stage is usually uniform and fast.

2. Falling rate drying period

As the drying progresses, the surface moisture gradually decreases, and internal moisture begins to migrate to the surface. The drying rate starts to decline. This stage is controlled by the migration and evaporation of moisture from the interior to the exterior of the grain, with the moisture gradient gradually decreasing and the drying rate significantly slowing down. Over time, the imbalance in temperature and moisture gradients may lead to uneven drying, especially in larger grain particles where the unevenness becomes more pronounced. The rate of decline in drying speed during this phase depends on the ease with which internal moisture migrates; typically, as moisture decreases, the drying rate declines exponentially.

3. Residual drying period

In the final stage, the remaining moisture within the grain is almost completely evaporated, and the drying rate becomes very low. At this point, evaporation of moisture is primarily driven by extremely small moisture gradients, causing the drying rate to flatten out. The duration of this stage is relatively long, and in some cases, continued drying may result in damage to the grain’s quality, especially at higher temperatures. In this phase, the decline in drying rate is primarily due to the near-zero moisture gradient, which significantly reduces the driving force for moisture migration.

The drying curve is a key tool for describing these stages. It illustrates the variation in moisture content over time during the drying process, reflecting the characteristics of different drying phases. During the constant rate drying period, the curve remains flat, indicating that the evaporation rate of moisture is constant. In the falling rate drying period, the curve gradually slopes downward, showing a decline in the drying rate. Finally, during the residual drying period, the curve becomes flat, with the rate of moisture reduction becoming very low.

The drying curve is typically represented as the relationship between moisture ratio or moisture content (*X*) and time. The curve usually displays three distinct stages:

Moisture content (*X*):

(9) $$X=\frac{m_{w}}{m_{s}}\times 100$$

where: *m*_*w*_ is the mass of water in the grain (kg). *m*_*s*_ is the mass of dry solid grain (kg). *X* is the moisture content (%).

Moisture ratio (MR):

(10) $$MR=\frac{X_{t}-X_{e}}{X_{0}-X_{e}}$$

where: *X*_*t*_ is the moisture content at time *t* (kg water per kg dry solid). *X*_*e*_ is the equilibrium moisture content (kg water per kg dry solid). *X*_0_ is the initial moisture content (kg water per kg dry solid).

Falling rate drying equation:

(11) $$\frac{dX}{dt}=-n(X-X_{e})$$

where: $\frac{dX}{dt}$ is the drying rate (kg/s). *n* is a constant related to the material’s properties and drying conditions. *X* is the moisture content at time *t*. *X*_*e*_ is the equilibrium moisture content.

These formulas help quantify the moisture migration dynamics during different drying stages and provide a mathematical description of the actual drying process.

**Figure 2:**
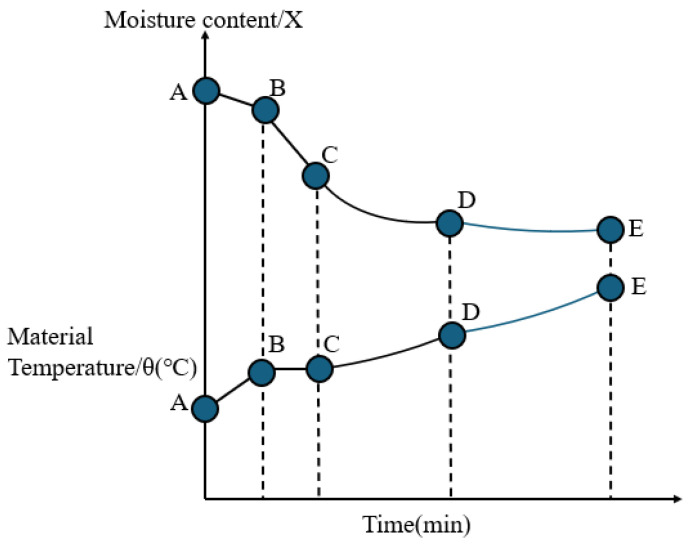
A typical drying curve. A,B: Preheating stage. The material absorbs heat to increase the vaporization rate, but the moisture content and material temperature change little. B,C: Constant rate drying stage. The material temperature remains constant, and the change in moisture content is relatively steady. C,D: Falling rate drying stage. The material begins to increase in temperature, and the change in moisture content slows down. D,E: Residual drying stage.

A typical drying curve is often represented as follows (Figure 2).

### 2.3. Changes in Grain Physical Properties

During the grain drying process, significant changes occur in the physical properties of the grain, particularly in parameters like moisture content, thermal conductivity, and density [36]. These changes influence the efficiency of the drying process and the final quality of the grain [37].

(1) Moisture content

Moisture content is the primary parameter used to assess the water content of grain, which decreases as the drying process progresses. Moisture content is typically expressed as a percentage by mass (%). The effect of moisture content on the drying rate is crucial. At the initial stages, high moisture grains have higher thermal conductivity and faster drying rates, but as the moisture content decreases, the drying rate gradually slows. Therefore, the variation in moisture content during the drying process not only affects the drying rate but also significantly influences the final quality of the grain. For grain drying to be considered effective and properly conducted, the target moisture content should typically be reduced to around 12–14%. This moisture range ensures that grains are adequately dried for storage, minimizing the risk of mold growth and spoilage.

Moisture content (*X*) is a key parameter in drying. The moisture ratio is often used to quantify moisture content changes during drying. The moisture ratio is given by Equation (10). The moisture content (*X*) is given by Equation (9).

(2) Thermal conductivity

Thermal conductivity refers to the ability of a material to conduct heat. The thermal conductivity of grain changes with moisture content. Typically, as moisture decreases during drying, the thermal conductivity also decreases. Higher moisture content generally means higher thermal conductivity, which aids in faster heat transfer and accelerates the drying process. However, as moisture content decreases, the thermal conductivity of the grain drops, leading to a reduction in heat transfer efficiency and consequently slowing the drying rate. Therefore, temperature control during the drying process is critical to ensure effective heat transfer [38].

(3) Density

Density is another important physical parameter that affects the drying and mechanical processing of grain. When moisture content is high, the density of the grain is relatively low. As moisture evaporates, the volume and mass of the grain gradually decrease, leading to an increase in density. The variation in density not only affects air flow and heat exchange efficiency during drying but may also have an impact on mechanical damage or cracking of the grain. Therefore, the changes in density during drying need to be carefully considered [39].

### 2.4. Effects of Drying on Grain Quality

The drying process has a profound impact on the quality of grains, particularly in terms of nutritional content, germination rate, and sensory properties [40]. To ensure that grains maintain good quality after drying, precise control of temperature, humidity, and drying time are essential during the process [41,42].

(1) Changes in nutritional content

High-temperature drying can lead to the loss of heat-sensitive nutrients in grains, such as B vitamins, vitamin C, and antioxidants [43]. As the drying temperature increases, the structure of components such as proteins, carbohydrates, and fats in the grain may change, thereby affecting its nutritional value [44]. For example, high-temperature drying can cause the degradation of cellulose, minerals, and proteins in wheat bran, reducing its nutritional value [45,46].

(2) Changes in germination potential

The germination rate is an important indicator of the quality of grain seeds. During the drying process, excessive heat or over-drying can impair the physiological functions of the grain seeds, resulting in a decrease in germination ability. Particularly under high temperatures or rapid drying conditions, the cell structure of the grain may be damaged, leading to a significant reduction in germination rate. Therefore, gentle drying and temperature control are critical to maintaining the germination capacity of grains [40,47].

(3) Sensory quality

The sensory quality of grains includes appearance, taste, texture, and other aspects. Over-drying can cause grains to become hard, lose their luster, or even develop cracks and damage, negatively affecting their market value and consumer acceptability. Thus, it is important to balance the drying rate with the sensory quality of the grain, ensuring that its appearance and taste remain in their optimal state.

## 3. Main Grain Drying Technologies

### 3.1. Hot Air Drying

**Figure 3:**
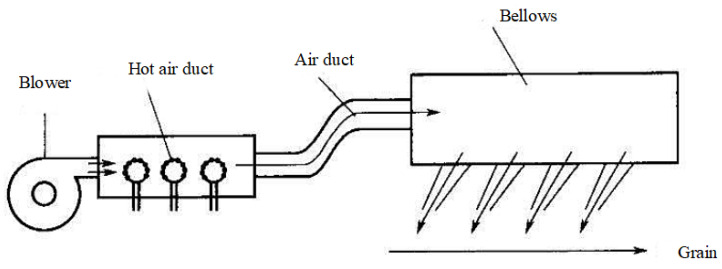
Basic principle diagram of hot air drying.

Hot air drying (HAD) is one of the most commonly used grain drying technologies. The basic principle involves contact between hot air and grains, where the thermal energy from the hot air is used to evaporate moisture from the grains. The typical temperature range for hot air drying is 40–90 °C, depending on the type of grain and the required drying rate. Hot air drying is widely applied in the drying of agricultural products such as grains and fruits, due to its efficiency and simplicity of operation. However, hot air drying often faces challenges such as low drying efficiency, high energy consumption, and quality degradation of the products [48,49]. Therefore, optimizing the design of the hot air drying process is crucial [50]. Figure 3 shows a basic principle diagram of hot air drying. As shown in the figure, the hot air is heated by the heater and then enters the drying chamber, where it comes into contact with the material to be dried, removing the moisture from the material. Finally, the humid air is expelled through the exhaust system [51].

#### 3.1.1. Continuous and Batch Hot Air Drying

The application modes of hot air drying can be classified into continuous drying and batch drying. Both drying methods have their respective advantages and disadvantages, making them suitable for different production scales and requirements.

**Figure 4:**
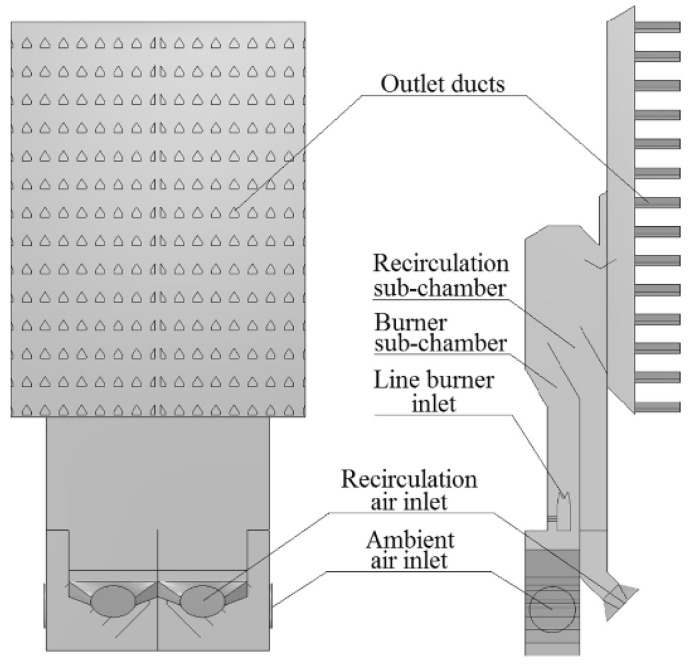
Geometry of the hot air chamber [52].

In continuous drying systems, grain materials are continuously fed through the drying chamber while hot air constantly flows in, allowing moisture to evaporate steadily from the grains. This method is suitable for large-scale industrial production and can achieve high production efficiency. According to the study by Kjær et al., (2018) [52], continuous drying offers a high level of automation and stable drying rates, making it especially suitable for large-volume, standardized drying operations. One example of the geometry of the hot air chamber is shown in Figure 4. It represents one of several designs in use, which vary significantly in their characteristics [52]. Furthermore, Mayta and Massarani et al., suggested that continuous drying systems incorporate innovative heat recovery systems and air circulation equipment in their design, enhancing the system’s energy efficiency and drying performance [53].

Batch drying refers to a process where grains are charged into the dryer and heated continuously for a specific period. During this period, the grains are exposed to hot air, allowing moisture to evaporate. After the heating phase, the grains are cooled and/or discharged [54]. This method is typically used for smaller-scale production or laboratory studies. The advantage of batch drying is that it allows for more precise control over drying conditions, such as temperature and humidity, making it suitable for high-quality, low-volume grain products. Delfiya et al., (2022) noted that batch drying systems are better at preserving the appearance and nutritional content of grains, especially when handling products with higher added value [55]. Additionally, Singh et al., (2020) demonstrated that the solar-assisted heat pump drying system (SAHPD), which utilizes the characteristics of batch hot air drying, not only improves drying efficiency but also enhances overall economic benefits by optimizing energy consumption [56].

#### 3.1.2. Hot Air Circulation System Design

Hot air circulation system design in hot air drying systems is a key factor that influences drying efficiency and energy consumption [57]. An effective circulation system ensures the even distribution of hot air within the drying chamber, reducing temperature and humidity differences, thereby improving both the efficiency and uniformity of the drying process [58]. The key components of the hot air circulation system typically include air ducts, fans, heat exchangers, and temperature and humidity control devices. In an efficient circulation system, hot air continuously circulates through the drying chamber, coming into contact with the grain surface [59]. The circulation design must ensure that air flows evenly through all areas of the drying chamber, avoiding hot spots or areas with insufficient drying. By using variable speed fans and airflow controllers, airflow speeds can be adjusted based on the moisture content of the grain and the required drying rate, thus maintaining optimal drying conditions [60]. Heat recovery systems, such as air-to-air heat exchangers, can further improve energy efficiency by recovering and reusing heat from the exhaust air, reducing the need for additional heating energy. The integration of smart controls and sensors allows for real-time monitoring and automatic adjustment of temperature, humidity, and airflow, optimizing the overall drying process [61].

**Figure 5:**
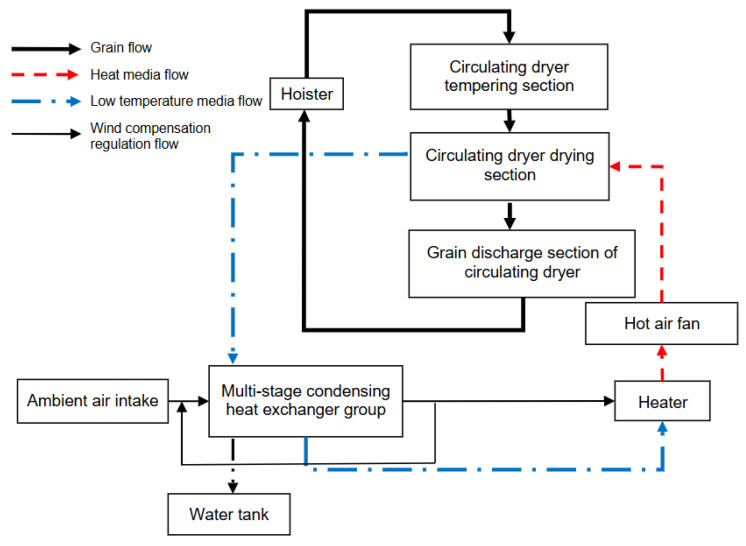
Schematic diagram of a drying system [64].

In the hot air drying process, the flow path and circulation mode of hot air have a significant impact on the drying effect. According to the study by Wang et al., (2020), using efficient airflow design can achieve uniform air distribution, reduce hot spots, and improve drying efficiency [62]. The hot air circulation and temperature control system proposed by Bie et al., (2017) effectively controls the temperature and humidity during the drying process, ensuring the stability of the drying effect [63]. Furthermore, Wang et al., (2022) [64] proposed an energy-efficient grain circulation drying system that uses clean electric energy as a heat source while recycling exhaust gas, resulting in significant energy savings. The system demonstrated a 44% reduction in energy consumption compared to conventional methods, highlighting the potential for improved energy efficiency and environmental protection in the drying process [64]. The design of the drying system is illustrated in Figure 5.

Hot air drying systems are typically equipped with heat exchangers to recover part of the heat from the exhaust air, thereby reducing energy consumption and improving thermal efficiency. Delfiya et al., (2022) demonstrated that heat recovery technology can improve system efficiency by approximately 25% and effectively reduce drying costs [55]. Mayta and Massarani et al., found that recovering waste heat from exhaust gases and reheating fresh air not only reduces energy consumption but also improves the system’s thermal efficiency [53].

#### 3.1.3. Energy-Efficient Control Strategies

With the growing demands for energy consumption reduction and environmental protection, improving the energy efficiency of hot air drying and implementing intelligent control systems have become key areas of research [65].

Energy efficiency optimization in the hot air drying process is mainly achieved through improvements in heat source design, heat recovery systems, and air flow paths. Wang et al., (2022) pointed out that the use of energy-efficient fans and optimized heat exchange technology can significantly reduce energy waste in hot air drying and improve energy efficiency [64]. Additionally, Wang et al., (2020) proposed the use of an exhaust gas recovery system, which recovers heat from the exhaust air and reheats fresh air, thus reducing the demand for external heat sources [62]. Research by Majumder et al., (2022) also showed that the combination of solar-assisted systems and heat recovery technologies can significantly enhance the overall energy efficiency of drying systems [66].

With the advancement of computational technologies, intelligent control systems have been applied in hot air drying to increase the automation and precision of the drying process. Delfiya et al., (2022) developed a drying control system based on fuzzy control and PID algorithms, which can automatically adjust temperature and humidity based on real-time moisture changes in the grains, thereby optimizing drying performance and reducing energy waste [55]. Moreover, Wang et al., (2022) proposed using an Internet of Things (IoT)-based real-time monitoring system that not only enables real-time moisture content measurement but also dynamically adjusts drying parameters to achieve optimal drying results [64].

Studies have shown that control strategies for hot air drying primarily include temperature control, humidity control, and airflow control. Wang et al., (2022) demonstrated that, by combining real-time feedback from environmental temperature, humidity, and grain moisture content, adaptive control strategies can effectively reduce energy consumption and improve drying rates [64]. Bie (2017) further emphasized the importance of temperature and airflow control, noting that precise airflow regulation can reduce energy losses in hot air and improve drying efficiency [63].

### 3.2. Microwave Drying

**Figure 6:**
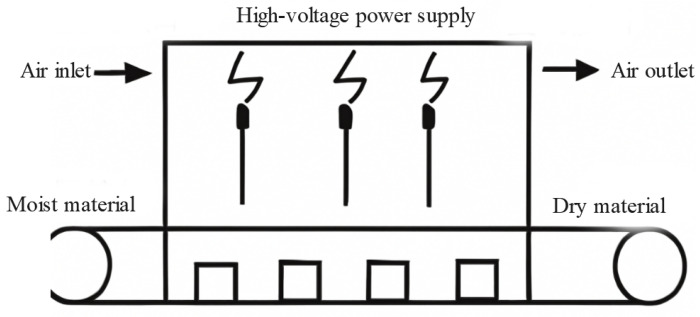
Principle diagram of the microwave drying system.

Microwave drying (MWD) technology has demonstrated many advantages in grain drying, such as rapid heating, uniform heating, and efficient energy conversion, leading to its widespread research and application in recent years [67,68]. However, practical applications still face challenges, including insufficient energy efficiency and high equipment costs. The temperature for microwave drying is typically controlled between 50–85 °C. Despite the significant benefits of microwave drying, practical applications still face challenges, including insufficient energy efficiency and high equipment costs. Therefore, researchers have conducted a series of optimizations and explorations to improve the performance of microwave drying, particularly in its application to grain drying. The following text will discuss in detail the heating mechanism, penetration ability, energy efficiency improvements, and the combined application of microwave drying with other drying technologies in grain drying [69]. Figure 6 illustrates the principle of microwave drying.

#### 3.2.1. Heating Mechanism and Penetration Ability

The core principle of microwave drying is the interaction of microwave radiation with water molecules in the material, which excites the water molecules to undergo polar rotation and generate frictional heat, causing rapid moisture evaporation. Unlike traditional hot air drying methods, microwave drying has a significant internal heating characteristic, allowing moisture to evaporate quickly from within the material, thereby improving drying efficiency [70].

During microwave drying, water molecules rotate due to the influence of the electromagnetic field, generating friction and releasing heat. Microwave heating can create a uniform heating effect within the material, reducing temperature differences and enhancing heating efficiency. According to the study by An et al., (2024), microwave heating technology can effectively reduce temperature differences in grains, thereby accelerating the moisture evaporation process [71]. However, the penetration depth of microwave energy is limited, making it typically suitable for thin layers of material or grains with high moisture content. For thicker materials or those with lower moisture content, the penetration ability and heating effect of microwaves may be restricted.

The penetration ability of microwaves depends on their frequency, the moisture content of the material, and the dielectric properties of the material. Generally, the penetration depth of microwaves decreases with increasing frequency. For most grains, the penetration depth of microwaves typically reaches a few centimeters, meaning that microwave heating is usually concentrated on the surface and near-surface regions of the material [10,72]. However, when dealing with thicker materials, the microwave heating effect gradually weakens. Therefore, supplementary measures, such as the combined application of microwave and hot air drying, are needed to improve penetration depth.

#### 3.2.2. Drying Uniformity and Energy Efficiency Issues

A key challenge in microwave drying is improving the uniformity and energy efficiency of the drying process. While microwave heating can generate relatively uniform heat distribution within the material, the inherent non-uniformity of the microwave field may still lead to localized overheating or uneven drying, which can affect the drying outcome and the final quality of the grains.

During microwave drying, the distribution of heat is often uneven, with some areas experiencing overheating or lower temperatures, which leads to non-uniform drying [73]. To address this issue, researchers have proposed various optimization solutions, such as using rotating devices, microwave waveguides, or reflectors to optimize the microwave field distribution [74,75]. Additionally, Khodabakhshi et al., (2015) noted that proper material flipping or stirring can effectively prevent localized overheating and promote uniform drying [72].

Although microwave drying has relatively high energy conversion efficiency, in practical applications, uneven heat absorption by the material can still lead to energy loss. To improve energy efficiency, researchers have proposed various strategies, including power regulation, multi-stage drying, and optimized microwave power scheduling [13,76]. These methods help reduce energy waste and further enhance the energy efficiency of microwave drying [77]. Additionally, studies have shown that, through proper drying temperature control and power distribution, energy loss can be minimized to the greatest extent.

#### 3.2.3. Combined Drying with Other Technologies

Although microwave drying technology has significant advantages, it also has limitations, such as uneven heating, limited penetration ability for thick materials, and the potential for surface damage due to excessively rapid drying. Therefore, many researchers have explored the combined application of microwave drying with other drying technologies to leverage the complementary advantages of each, further enhancing the drying effectiveness and improving the quality of the grains [78,79].

The combination of microwave and hot air drying can effectively compensate for the shortcomings of microwave drying in terms of heating uniformity and penetration depth. Microwaves quickly heat the material’s surface and evaporate a large amount of moisture during the initial drying stage, while hot air helps remove the evaporated water vapor from the surface, preventing moisture from re-entering the material. According to the study by Wang et al., (2021), the combined use of microwave and hot air drying can maintain a high drying rate while preventing surface cracking and localized overheating of the material [13].

The combination of microwave and vacuum drying has also received widespread attention [80]. The vacuum environment helps reduce the drying temperature and prevents high temperatures from negatively impacting the material’s quality, while microwave drying accelerates moisture evaporation. Tepe et al., (2022) found that the combined use of microwave and vacuum drying significantly improved the drying efficiency of grains, effectively reduced energy consumption, and better preserved the material’s nutritional content and flavor [77].

The combined application of microwave and freeze drying is particularly suitable for heat-sensitive materials, as it can increase the drying speed while maintaining drying quality. Through freeze pretreatment, the moisture in the material is converted into ice crystals, and microwave drying accelerates moisture removal through sublimation. Khodabakhshi et al., (2015) found that the combination of microwave and freeze drying allows for efficient drying at low temperatures, thereby reducing the damage to the nutritional content of grains caused by high temperatures [72,81].

The combined application of microwave and infrared drying enhances drying efficiency and optimizes energy use. Microwave heating penetrates the material deeply, heating the interior through the vibration of water molecules, while infrared primarily heats the surface, rapidly increasing the temperature. Mukwevho and Emmambux (2022) [82] found that the combined treatment of infrared and microwave effectively promotes starch pre-gelatinization and protein denaturation, thereby altering the functional properties of the resulting flour and reducing viscosity. This combined approach, through the complementary effects of both heating mechanisms, not only accelerates the drying process but also preserves the nutritional content of the grain, making it particularly suitable for drying heat-sensitive materials [82].

### 3.3. Infrared Drying

**Figure 7:**
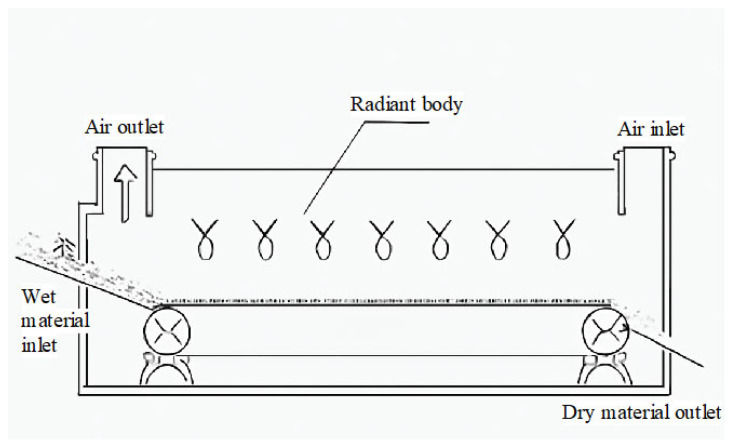
Basic principle diagram of the infrared drying process.

Infrared drying technology uses infrared radiation to heat the surface of the material by exciting the vibrations of water molecules [83], which accelerates moisture evaporation and enables rapid and uniform heating of the material [84,85]. The typical temperature range for infrared drying is 40–80 °C, but it has limited penetration depth, so it is often combined with other drying technologies for better performance. This technology is suitable for materials with high moisture content and operates at lower temperatures, helping to retain the nutritional content and color of the material. Due to its limited penetration depth [86], infrared drying is typically combined with other drying technologies to improve its effectiveness [87]. It is especially suitable for organic grains, functional grains, and grain products, effectively controlling the drying process and reducing quality loss [88]. The basic principle diagram of the infrared drying process is shown in Figure 7.

#### 3.3.1. Surface Heating Principle

The core principle of infrared drying technology is to use infrared radiation to directly heat the surface of the material, causing the moisture on the surface to evaporate rapidly. Infrared radiation can be classified into near-infrared (0.75–1.5 μm), mid-infrared (1.5–5 μm), and far-infrared (5–1000 μm) based on wavelength [89]. Near-infrared and mid-infrared radiation have higher energy, making them suitable for materials with higher moisture content. Kotov (2020) proposed that infrared radiation excites the vibration of water molecules, causing them to absorb infrared energy and accelerate moisture evaporation [90]. Since the effect of infrared radiation is primarily concentrated on the surface of the material, the drying process is fast, significantly shortening the drying time [90]. Palamarchuk et al., (2021) [91] further found that infrared radiation transfers heat to the material’s surface, and through heat conduction, the heat is transferred to the interior of the material, ultimately achieving moisture evaporation. However, due to the weak penetration ability of infrared radiation, it typically cannot effectively penetrate the interior of the material. Therefore, infrared drying is more suitable for materials with higher surface moisture [91]. For drying moisture within the material, Kalinichenko et al., (2024) pointed out that other heating methods, such as hot air drying, are usually needed to improve the drying effect [92].

Additionally, due to the weak penetration of infrared radiation, researchers have proposed using multi-band infrared radiation sources in combination with rotation or stirring techniques to ensure uniform heating of the material’s surface and further improve drying efficiency. Nascimento et al., (2019) demonstrated through experiments that multi-band infrared radiation sources can effectively improve drying uniformity and prevent localized overheating [93]. Timm et al., (2020) also noted that using multi-band infrared radiation can excite water molecules at different wavelengths, enhancing overall moisture evaporation efficiency and demonstrating excellent drying performance when applied to different materials [94].

#### 3.3.2. Application Range

Infrared drying technology, due to its high efficiency and energy-saving characteristics, is particularly suitable for drying high-value-added grains, such as organic grains, functional grains, and grain products. These grains have strict requirements for temperature and time control during the drying process, and infrared drying can precisely adjust the drying conditions to effectively meet these demands.

**Figure 8:**
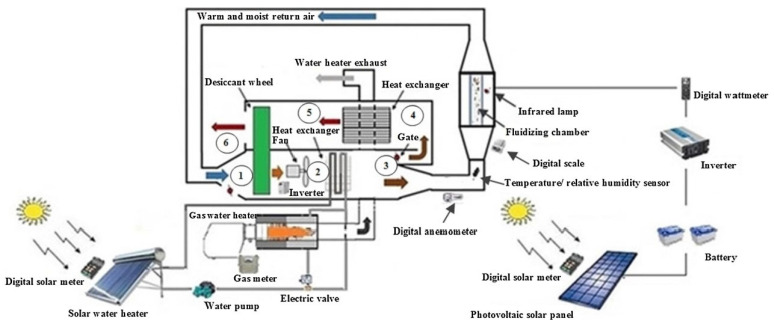
A solar-assisted fluidized bed dryer driven by solar energy with an infrared lamp [95].

Organic grains, which have not been chemically treated, typically have higher moisture content, and traditional hot air drying methods can lead to the loss of nutritional components and color. Mehran et al., (2019) pointed out that infrared drying can effectively preserve the nutritional content and color of organic grains through shorter drying times and lower operating temperatures, while avoiding over-drying and high-temperature damage [95]. Figure 8 shows the design of a solar-assisted fluidized bed dryer driven by solar energy with an infrared lamp, as designed by Mehran et al. The system uses solar energy as the primary heat source and employs an infrared lamp to enhance drying efficiency. Additionally, Jittanit and Srzednicki (2010) also indicated that infrared drying is particularly suitable for removing surface moisture from organic grains, preventing the nutrient loss typically caused by high temperatures in traditional drying methods [96]. Therefore, infrared drying can significantly improve the drying efficiency of organic grains while preserving their high-quality nutritional and sensory characteristics.

Functional grains, such as those high in protein and fiber, are prone to nutrient loss during drying due to overheating. Timm et al., (2020) pointed out that infrared drying can maximize the retention of the nutritional value and bioactivity of functional grains without exceeding their thermal stability limits [94].

In the production of grain-based products, such as instant cereals and grain powders, infrared drying can precisely control the moisture content of the product, avoiding the over-drying and nutrient loss commonly seen with traditional drying methods. Okeyo et al., (2017) demonstrated in their study that infrared drying effectively controls the drying process of instant cereals, preserving their flavor and texture [97]. Jittanit and Srzednicki (2010) pointed out that infrared drying can prevent common issues in traditional drying methods, such as dull color and flavor loss, making it particularly suitable for the production of high-quality grain-based products [96].

Further research by Mehran et al., (2019) found that infrared drying not only improves drying efficiency in the production of instant cereals but also preserves higher levels of nutritional content and flavor, giving these products a competitive advantage in the market [95]. Through drying experiments on different types of grain-based products, the researchers demonstrated the significant advantages of infrared drying in retaining the flavor and nutritional value of grain products.

#### 3.3.3. Energy Efficiency

Infrared drying technology is widely used in the drying of various materials, due to its high energy conversion efficiency. Compared to traditional hot air drying, infrared drying directly transfers infrared radiation energy to the material surface, reducing heat loss in the air, thus allowing for more efficient energy utilization. The heating method of infrared radiation results in higher heating rates and lower operating temperatures, which helps achieve better drying results in a shorter time, further reducing energy consumption.

In addition, infrared drying systems typically have high thermal energy conversion efficiency, enabling rapid heat transfer and concentrated use of energy. This is particularly noticeable in the drying of high-moisture materials. Studies have shown that infrared drying can effectively save energy, especially when processing high-value-added grains and functional grains, as it reduces energy consumption while maintaining quality. Nascimento et al., (2019) pointed out that the use of multi-band infrared radiation sources not only improves drying efficiency but also optimizes energy distribution, reducing unnecessary energy waste [93].

However, the energy efficiency of infrared drying is also influenced by several factors, such as the properties of the material, the humidity of the drying environment, and the wavelength of the radiation source. To further improve the energy efficiency of infrared drying, researchers are exploring the integration of other energy-saving technologies, such as heat recovery systems or solar-assisted drying technologies, to reduce energy consumption and enhance the overall sustainability of the system. By optimizing the design of radiation sources and improving the control strategies of the drying process, infrared drying technology is expected to play an important role in energy conservation, emission reduction, and lowering production costs.

### 3.4. Freeze Drying

**Figure 9:**
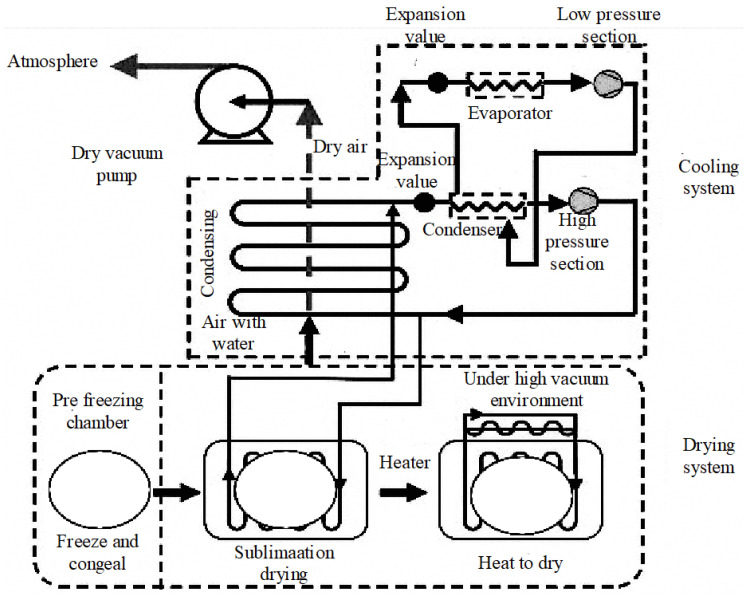
Freeze drying structural principle diagram.

**Figure 10:**
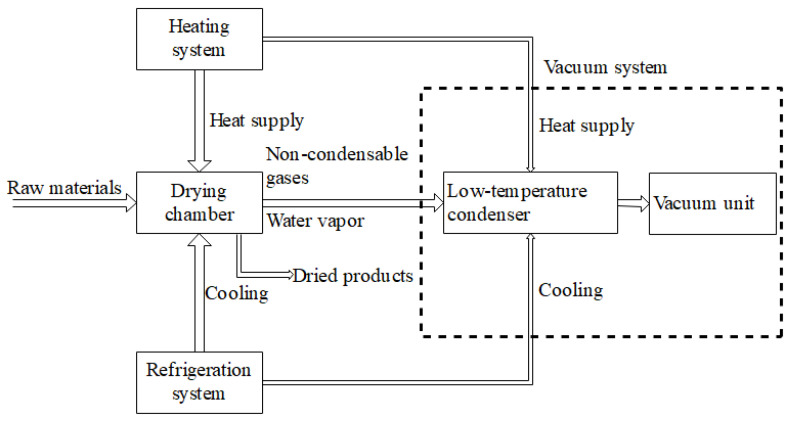
Freeze drying system principle diagram.

Freeze drying (FD) is a drying technology that removes moisture by freezing the material to extremely low temperatures and then sublimating the moisture under vacuum conditions. In recent years, freeze drying has demonstrated significant advantages in preserving the nutritional components and sensory quality of food, particularly in retaining heat-sensitive ingredients such as vitamins and minerals. Although the equipment and operational costs are relatively high, its application has gradually increased, especially in the preservation of high-value-added grains and seeds, yielding good results in practice. To better illustrate the working mechanism of the freeze drying process, Figure 9 and Figure 10 present the structural principle diagram of the freeze drying system. This diagram clearly outlines the key components and their interconnections, including the vacuum unit, cooling device, heat exchanger, and low-temperature condenser, which are essential for understanding the operational principles of the freeze drying process.

The freeze drying system is a drying process that removes moisture through sublimation to preserve the material. The raw material is first frozen and then, under vacuum conditions, it is heated to directly sublimate the ice into water vapor, avoiding the liquid phase [98]. The typical temperature range for freeze drying is generally kept between −40 °C and −10 °C to avoid degradation of heat-sensitive components. This process helps to preserve the material’s structure, nutritional components, and sensory quality. However, a significant disadvantage of freeze drying is its high energy consumption. The process requires substantial energy for both freezing and sublimation under vacuum conditions, making it less energy-efficient compared to other drying methods [99]. The system includes a heating system that provides heat, a refrigeration system that maintains low temperatures, a vacuum system that reduces pressure, and a low-temperature condenser that condenses the water vapor for removal. The coordinated operation of these systems ensures efficient and effective freeze drying, making it particularly suitable for heat-sensitive products [100].

#### 3.4.1. Applications and Advantages of Freeze Drying Technology in Food Preservation

In terms of retaining heat-sensitive components, Mujumdar et al., (2016) [101] studied the retention of vitamin C and flavonoids, among other antioxidants, in fruits and vegetables during freeze drying. The results indicated that freeze drying is highly effective in preserving these water-soluble vitamins and antioxidants, with minimal changes in these components during storage. Compared to traditional hot drying methods, freeze drying demonstrated significant advantages in reducing the loss of heat-sensitive components [101]. Additionally, Desai (1964) pointed out that freeze drying significantly reduces the degradation of proteins and minerals, thereby preserving the original nutritional components of food [102].

In terms of antioxidant activity and flavor preservation, freeze drying also demonstrates significant advantages. Ivančević et al., (2012) [103] and Ma et al., (2022) [104] found that freeze drying effectively preserves the natural color and aroma components in raspberries and other fruits, while these components undergo significant degradation in other drying methods. By avoiding high-temperature treatments, freeze drying maximizes the retention of volatile compounds and sensory characteristics in food.

In terms of texture and moisture retention, Lozano et al., (2014) [105] demonstrated that freeze drying not only preserves the original texture of grains but also maintains their moisture state over extended periods, effectively enhancing storage stability. Freeze drying preserves the original tissue structure of grains and seeds, making it particularly suitable for foods that require long-term storage and rehydration [105].

#### 3.4.2. Applications and Challenges of Freeze Drying Technology in High-Value Foods and Seed Preservation

Despite the significant advantages of freeze drying in preserving nutrition and sensory quality, its high equipment costs and complex operational processes have limited its widespread application. The freeze drying process not only requires a high initial investment but also involves complex steps and long processing times. As a result, freeze drying is primarily applied to high-value foods that have extremely high requirements for quality and nutrition, particularly demonstrating unique advantages in the preservation of specialty grains and seeds.

Freeze drying is particularly important in the processing of high-value-added grains, which often contain higher levels of proteins, vitamins, minerals, and other functional components. For example, Umuhozariho et al., (2020) [106] found that freeze drying better preserves the nutritional components in pumpkin powder, especially in terms of vitamin C and total carotenoids. Compared to traditional oven drying methods, freeze drying effectively reduces the loss of nutrients [106]. Bui et al., (2018) [107] studied the impact of freeze drying on rice and found that freeze dried rice is particularly ideal for applications requiring long shelf-life and rapid rehydration, such as emergency foods and military rations. The study showed that freeze drying significantly preserves the texture and rehydration properties of both parboiled and non-parboiled rice, offering a quality superior to traditional drying methods [107].

Freeze drying technology is equally crucial in seed preservation. Compared to other drying methods, freeze drying can effectively extend the storage period of seeds, while preserving their physiological viability and germination capacity. Pinto et al., (2024) demonstrated that freeze drying not only effectively extends the shelf life of strawberry seeds but also preserves their nutritional components and sensory characteristics, allowing these seeds to maintain a high germination rate even after long-term storage [108]. Additionally, Lee et al., (2024) further emphasized that freeze drying effectively prevents physiological damage during seed preservation, which is critical for maintaining a high germination rate [109]. In contrast, traditional drying methods, due to the high temperatures involved, can easily lead to changes in the physiological state of the seeds and a decrease in germination rate.

Freeze drying is also widely applied in the processing of high-value products that require extremely high standards for flavor and nutritional components, particularly in the fields of instant cereals and functional foods. Singh et al., (2024) pointed out that freeze drying effectively preserves the natural flavor and color of food, and when processing functional grains containing sensitive ingredients, it can effectively prevent the flavor and texture losses associated with other drying technologies [110]. Liaotrakoon et al., (2011) also noted that freeze drying can preserve the natural flavor of grains while avoiding the impact of thermal degradation on nutritional components, thereby enhancing the market competitiveness of these premium products [110,111].

Although freeze drying has irreplaceable advantages in the preservation and processing of many high-value foods, its high equipment and operational costs generally limit its widespread use to products with extremely high quality requirements and greater added value. The efficiency and high-quality assurance provided by freeze drying make it a highly promising technology in the food industry, particularly in areas such as high-value foods, seed preservation, and specialty grain processing. The future application prospects are vast [112].

### 3.5. Vacuum Drying

**Figure 11:**
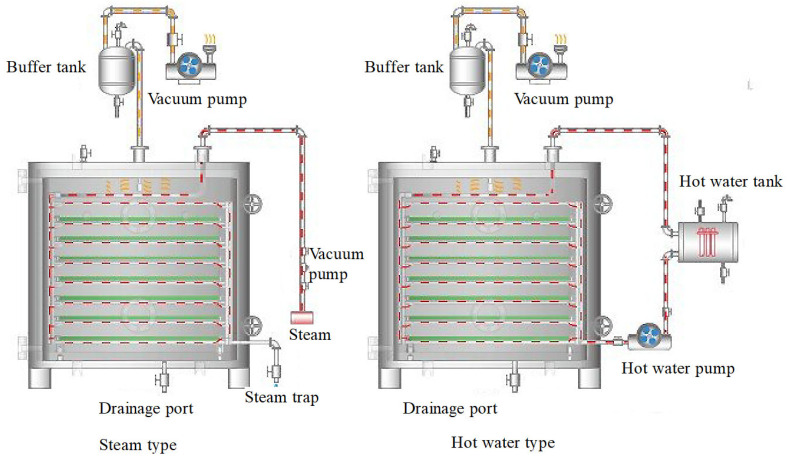
Principle diagram of rectangular vacuum drying [119].

The core principle of vacuum drying technology is to lower the air pressure to reduce the boiling point of water, allowing moisture to evaporate at lower temperatures and avoiding the damage caused by traditional high-temperature drying methods [113,114]. The temperature range for vacuum drying is typically between 30–60 °C, depending on the vacuum level and drying rate. In a vacuum environment, the material is typically placed in a sealed chamber under lower-than-normal atmospheric pressure [115]. Heat is transferred through a heating medium (such as hot water or steam) to facilitate the evaporation of moisture [116]. Figure 11 presents the principle diagram of the rectangular vacuum drying system, which illustrates the key components and their interconnections within the drying process. This diagram provides a comprehensive overview of how the system operates, including the integration of the buffer tank, vacuum pump, solenoid valve, and steam trap, among other critical elements. Since water can evaporate at low temperatures, this method is particularly suitable for temperature-sensitive materials, such as grains and seeds. Adamchuk and Shvidia (2020) pointed out that, during the vacuum drying process, by controlling changes in temperature, humidity, and pressure, moisture can be evenly distributed from the interior of the material to the surface, avoiding excessive drying on the surface, thus effectively preserving the material’s nutrients and structure [117,118].

#### 3.5.1. Application Range and Advantages

**Figure 12:**
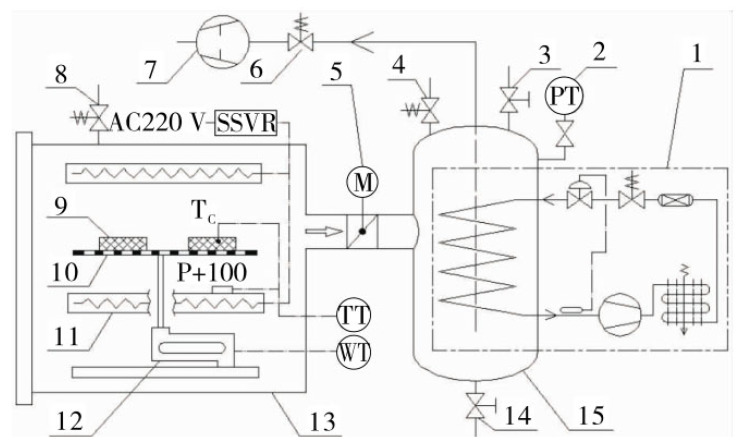
Schematic diagram of the drying system. 1. Cold trap refrigeration unit 2. Vacuum pressure transmitter 3. Hand valve 4. Exhaust valve 5. Electric butterfly valve 6. Oil stop valve 7. Vacuum pump 8. Gas valve 9. Material 10. Tray 11. Electric heating plate 12. Weight sensor 13. Vacuum chamber 14. Drain valve 15. Cold trap [122].

Vacuum drying technology is primarily suited for temperature-sensitive materials, especially in the drying process of high-value grains and seeds. For these high-value agricultural products, traditional hot drying methods often lead to pyrolysis reactions, resulting in nutrient loss, flavor degradation, and other issues. Zhang (2007) demonstrated that, compared to traditional hot air drying, vacuum drying can efficiently remove moisture at lower temperatures, maximizing the retention of nutrients, color, and flavor in grains [120]. Additionally, vacuum drying effectively prevents chemical reactions such as oxidation, thereby better preserving the quality of food. Bedoya-Corrales et al., (2018), in their study of Annatto seeds, found that vacuum drying can effectively preserve key components, such as bixin, at lower temperatures and pressures, ensuring the stability of these components during storage [121]. Li Jianguo et al., designed a cryogenic vacuum drying system that precisely controls the material temperature within a set cryogenic range during the vacuum drying process, preventing protein denaturation and tissue damage. The system showed stable operation and effective temperature control within the desired range. This system demonstrates promising application potential and provides a platform for future cryogenic vacuum drying research [122]. The schematic diagram of the drying system designed by these authors is shown in Figure 12.

#### 3.5.2. Disadvantages and Limitations

Despite its many advantages, particularly in preserving nutritional components and sensory characteristics, vacuum drying has some drawbacks, such as high equipment investment and relatively high operational and maintenance costs. The high cost of vacuum drying technology limits its application in large-scale production, especially for products with lower profit margins. Djordjević et al., (2023) pointed out that, although the initial investment in vacuum drying equipment is high, its application is widespread in high-value products, especially in the drying of agricultural products with extremely high quality requirements, such as specialty grains and organic crops, where it shows significant advantages [123].

#### 3.5.3. Energy Efficiency and Control Strategies

In terms of energy efficiency, vacuum drying has certain advantages as it operates at lower temperatures and pressures, reducing heat waste. However, to achieve higher energy efficiency and shorter drying times, precise control strategies are required during the vacuum drying process. Kozanoglu et al., (2006) found that vacuum drying reduces heat waste, especially when conducted under low-temperature and low-pressure conditions [124]. Furthermore, Jokić et al., (2012) [125] proposed that the control system of vacuum drying can optimize the drying process by adjusting parameters such as temperature, humidity, and pressure, thereby improving drying efficiency and energy usage efficiency. With this optimized control strategy, energy consumption can be reduced, while maintaining material quality and improving production efficiency [125].

### 3.6. Solar Drying Technology

With the advancement of renewable energy technologies and the increasing demand for green agriculture, solar drying technology, as a low-carbon and efficient method for grain drying, has gained widespread attention in both research and industry. This technology converts solar radiation into thermal energy through a collector, which then heats the drying air and conducts it into the drying chamber, facilitating the evaporation and migration of moisture from the grains. The typical temperature range for solar drying is 30–60 °C, depending on the intensity of solar radiation and environmental conditions. It is particularly suitable for rural areas, regions without grid electricity, or situations where energy access is difficult, offering advantages such as low operational costs, simple system structure, and environmental friendliness [126].

#### 3.6.1. Technical Principles and System Structure

A solar grain drying system typically consists of a collector, hot air delivery ducts, a drying chamber, a control system, and a thermal storage unit. Based on the energy transfer method and system configuration, it can be classified into the following three types:

Direct drying system: Grains are directly exposed to sunlight for drying. This method is simple but highly influenced by weather and sanitary conditions.

Indirect drying system: Air is heated through a collector and then introduced into the drying chamber for convective drying, effectively avoiding quality degradation caused by direct sunlight.

Hybrid drying system: This type of system combines solar energy with auxiliary energy sources (such as electric heating or butane gas) and incorporates a thermal energy storage system to enhance the continuity and stability of the drying process [127].

#### 3.6.2. Recent Research Progress

In recent years, solar drying technology has made significant breakthroughs in various areas, including the following:

(1) Structural design optimization

**Figure 13:**
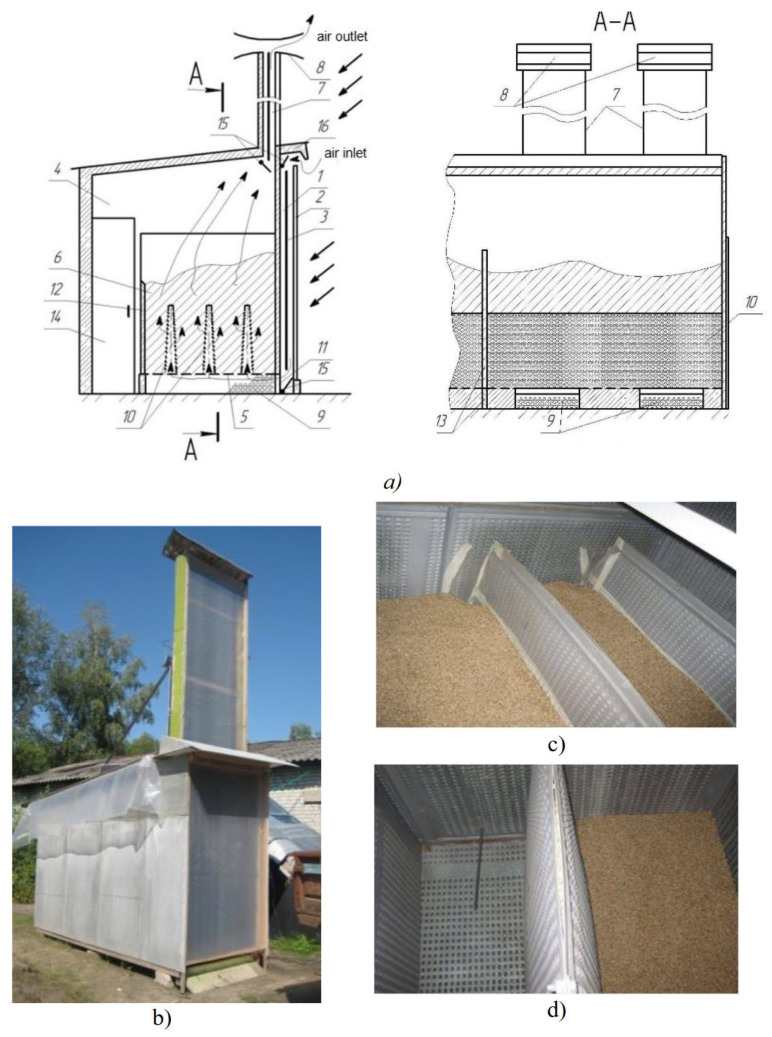
Grain storage with built-in solar drying system: (**a**) Basic diagram; (**b**) General view; (**c**) Grain storage sections; (**d**) Section view with temperature sensor. 1. Vertical solar collector 2. Translucent cover 3. Light-absorbing surface 4. Drying chamber 5. Perforated decking 6. Dried grain 7. Exhaust pipe 8. Deflector 9. Gravel accumulator 10. Perforated air-distribution channels 11. Openings in grain storage wall 12. Longitudinal partition 13. Transverse partition 14. Door 15. Shutters 16. Roof canopy [129].

Chavan et al., (2021) [128] used computational fluid dynamics (CFD) simulation technology to optimize the airflow structure and hot air recirculation path of solar dryers. They also conducted a systematic parametric study on fan placement, which improved drying uniformity and thermal efficiency [128]. Kupreenko et al., proposed a multi-stage heat collection system that improves drying speed and energy efficiency through the synergistic action of upstream and downstream heat sources. This system demonstrated high stability and thermal conversion efficiency under low-temperature and high-humidity conditions. The designed drying system is shown in Figure 13 [129]. Additionally, Shen et al., (2017) designed a solar drying system with phase change material (PCM) assistance, which effectively extended thermal energy storage time and improved the system’s drying efficiency and energy self-sufficiency [130].

(2) Control strategies and energy storage integration

To address the intermittent drying issue caused by fluctuations in solar radiation, Khalil et al., introduced a thermal energy storage system and combined it with an ON-OFF control strategy based on the ATmega microcontroller, successfully achieving continuity in the drying process. In practical operation, this system reduced the initial moisture content of the grain from 42.5% to about 16% within 10 h [125]. Moreover, Fagunwa et al., (2018) further developed a thermal energy storage chamber with valve regulation for heat supply during nighttime or cloudy conditions, preventing grain reabsorption of moisture and effectively extending the drying time, thus optimizing the system’s energy efficiency [131,132,133,134].

(3) System integration and comprehensive performance enhancement

To further improve drying efficiency, Senthil et al., (2021) proposed a solar drying system based on phase change materials (PCM), which effectively reduced drying time by 60% and improved product quality, while optimizing thermal efficiency and energy storage performance [135]. Asemu et al., (2020) [133] studied the drying characteristics of a novel solar bubble-type dryer, combined with thin-layer drying mathematical models, analyzing the drying process under different wind speeds and sample loads. They optimized drying time and energy efficiency, making it suitable for small farms and agricultural cooperatives [133].

### 3.7. Comparative Analysis of Grain Drying Technologies

Different grain drying technologies vary in terms of efficiency, cost, and the loss of active components. Each technology has its own advantages, limitations, and suitable applications. A detailed comparison is provided in Table 1. Below is a summary of the key points:

(1) Sun or Shade Drying:

Advantages: Most cost-effective method, suitable for low-value products.

Disadvantages: Low efficiency, highly dependent on weather conditions, requires significant labor and cannot guarantee hygienic conditions, making it vulnerable to pest infestations and environmental factors.

(2) Drying Rooms:

Advantages: Moderate efficiency and lower costs, suitable for small to medium-scale production.

Disadvantages: Uneven drying, environmental pollution, high energy consumption, leading to suboptimal product quality.

(3) Hot Air Drying:

Advantages: Widely used for agricultural products like grains and medicinal herbs, simple to operate, suitable for large-scale production.

Disadvantages: High energy consumption, uneven temperature and humidity, which can degrade product quality.

(4) Microwave Drying:

Advantages: High efficiency, rapid drying, especially for materials with high moisture content.

Disadvantages: High equipment cost, difficulty in controlling moisture content, risk of surface overheating, which may cause nutrient loss.

(5) Vacuum Drying:

Advantages: Ideal for temperature-sensitive and high-value products, minimizes nutrient loss, preserves flavor.

Disadvantages: High equipment cost, high energy consumption, longer processing time, limiting its use in large-scale production.

(6) Freeze Drying:

Advantages: Best for preserving nutrients and quality, especially for high-value products like fruits, vegetables, and seeds.

Disadvantages: High initial investment and maintenance costs, low energy efficiency, limiting large-scale use.

(7) Infrared Drying:

Advantages: High drying efficiency with uniform heating, ideal for materials with high surface moisture.

Disadvantages: Limited penetration depth, ineffective for thicker or lower moisture materials, often combined with other drying technologies to enhance performance.

(8) Solar Drying:

Advantages: Low cost, environmentally friendly, suitable for remote areas, low operating costs.

Disadvantages: Efficiency is weather-dependent (affected by cloudy days or insufficient sunlight), high initial installation cost, requires more complex designs.

## 4. Drying Equipment and Industrial Applications

In modern grain drying technology, the selection and development of drying equipment are crucial [136]. Different types of drying equipment vary in terms of operating principles, adaptability, and energy efficiency. Choosing the appropriate equipment based on the type of grain and production requirements is essential for improving production efficiency, reducing energy consumption, and maintaining product quality. This section will introduce several typical drying devices. This section classifies and discusses grain dryers based on the direction of airflow relative to the grain and their operating methods. Given the significant overlap between the classification of grain drying equipment by operating principles and the previously discussed grain drying technologies, this section will not repeat the related content.

### 4.1. Classification by Airflow Direction Relative to Grain Movement

(1) Crossflow dryers

The design of crossflow dryers is characterized by the fact that the direction of grain movement is perpendicular to the direction of the hot air flow. These dryers typically feature cylindrical or square tower structures with perforated screens. The advantages include simple manufacturing processes, easy installation, low cost, and high productivity. However, they suffer from poor drying uniformity, higher energy consumption, and difficulty in drying multiple types of grains simultaneously, which may affect the final grain quality [137].

(2) Mixed flow dryers

In mixed flow dryers, hot air passes through the grain layer at different angles, creating a mixture of counterflow and cocurrent flow, or a combination of crossflow, counterflow, and cocurrent flow. These dryers use a tower structure composed of triangular or pentagonal boxes arranged alternately, ensuring uniform hot air distribution, lower energy consumption, and suitability for drying various grain types. However, the structure is complex, manufacturing costs are high, and the grains at the four corners of the dryer may experience slower moisture reduction [138].

(3) Cocurrent flow dryers

In cocurrent flow dryers, the direction of grain movement aligns with the direction of the hot air flow. These dryers often combine funnel-type intake ducts with corner-type exhaust ducts in a tower structure, with multiple hot air pipes supplying different or similar hot air. The use of high-temperature hot air leads to low energy consumption and high production rates, making it ideal for drying grains and seeds with high moisture content. However, the manufacturing costs are high, and high-pressure fan power is required [139].

(4) Counterflow dryers

In counterflow dryers, the direction of grain movement is opposite to the direction of hot air flow. The temperature of the grains upon discharge is close to that of the hot air, and the moisture content of the grains balances with the hot air’s moisture. This design is suitable for cooling and ventilation of grains after drying, as it prevents overheating and damage to the grains [140].

(5) Mixed counterflow and cocurrent flow dryers

This type of dryer combines the advantages of cocurrent and counterflow dryers to achieve high-temperature, fast drying, increased drying capacity, and the maintenance of grain quality and uniform moisture content without increasing energy consumption [141].

### 4.2. Classification by Working Method

(6) Batch operation grain dryers

In batch operation dryers, drying begins with the lowest layer of grain and gradually progresses upwards. As the drying process continues, three layers form: the dried layer, the drying layer, and the undried layer. As the drying process progresses, these layers move upward. These dryers are flexible and suitable for small batch, multi-variety drying, but they have lower drying efficiency [142].

(7) Continuous operation grain dryers

Continuous operation dryers allow grains to flow continuously through the drying chamber, achieving continuous drying. These dryers offer high drying efficiency, making them suitable for large-scale drying operations, but they may be less adaptable to various grain types [143].

(8) Recirculation operation grain dryers

Recirculation operation grain dryers allow grains to circulate automatically in the dryer, ensuring uniform drying and maintaining good quality. This type of dryer includes closed-loop circulation dryers and split-loop circulation dryers, offering better drying uniformity. However, they are more complex in structure [144,145,146,147].

## 5. Challenges and Development Trends

Despite the considerable advancements in grain drying technologies, several challenges continue to impede their widespread application. Energy consumption remains a significant issue, as many drying methods, particularly traditional hot air drying, consume substantial amounts of energy, which is not only costly but also contributes to environmental degradation. The pressure to reduce carbon footprints is mounting, and more energy-efficient, eco-friendly drying technologies are essential. To address this, emerging drying methods such as microwave, infrared, and solar drying are gaining traction due to their energy-saving benefits. However, these methods still face practical hurdles, including high costs, inconsistent drying, and the challenges of maintaining the nutritional and sensory quality of the grains.

Another persistent challenge is uneven drying, which is a critical concern for large-scale operations. In conventional methods, the outer layers of the grain often dry faster than the interior, leading to over-drying or insufficient moisture removal. This inconsistency can affect both the quality and the storage stability of the grains. In addition, high-temperature drying can lead to significant nutrient degradation, particularly of sensitive vitamins and antioxidants, necessitating the development of more precise temperature control techniques that minimize this loss.

The future development of grain drying technology will need to focus on integrating multi-functional drying systems that combine different technologies to optimize energy use and drying efficiency. Hybrid systems, such as microwave-assisted hot air drying or solar-assisted infrared drying, hold promise in addressing both energy efficiency and quality retention issues. Moreover, the incorporation of smart systems, such as real-time monitoring and automated controls, will be crucial for optimizing drying processes and ensuring uniformity.

## 6. Conclusions

In conclusion, grain drying is an essential process that directly impacts the storage, transportation, and processing of grains. As global food security concerns intensify, the development of efficient, sustainable, and cost-effective grain drying technologies is crucial. Traditional methods such as hot air drying continue to dominate, but they present several challenges, including high energy consumption, slow drying rates, and quality degradation. New technologies such as microwave, infrared, and freeze drying offer significant advantages in terms of energy efficiency and nutrient preservation, but they also face limitations in terms of equipment cost and scalability.

The integration of advanced drying systems, including hybrid technologies and intelligent control mechanisms, appears to be the most promising direction for the future. As the demand for high-quality, sustainably produced grains increases, it will be vital for the industry to adopt these innovative solutions to improve efficiency, reduce energy consumption, and preserve the nutritional and sensory qualities of the grains.

Future research should focus on addressing the challenges of uneven drying, energy consumption, and quality degradation through the development of optimized drying systems and advanced control strategies. With ongoing technological advancements and the integration of smart, multi-functional systems, grain drying can evolve to meet the needs of modern agriculture, promoting both sustainability and food security.

## Figures and Tables

**Table 1:** Comparative analysis of grain drying technologies.

Drying Technology	Efficiency	Cost	Nutrient Loss	Other Aspects	Application Range	Advantages	Limitations
Sun or Shade Drying	Low	Low (Approx. USD 10–30/tonne)	Significant loss due to weather and pests	Greatly affected by weather, requires a lot of labor, poor hygiene, vulnerable to pests	Small-scale production, low-value grains	Lowest cost, simple to operate	Weather-dependent, low efficiency, no control over temperature and humidity, significant losses
Drying Room	Moderate	Low (Approx. USD 50–100/tonne)	Minimal	Uneven drying, pollutes the environment, requires high energy	Small and medium-scale production, grain products	Moderate drying efficiency, low cost, suitable for small drying operations	Uneven drying, environmental pollution, high energy consumption, fuel consumption
Hot Air Drying	Moderate	Moderate (Approx. USD 100–200/tonne)	Minimal	High energy consumption, slower drying speed, not suitable for high-value products	Widely used for medicinal herbs, grains drying	Suitable for large-scale production, easy to operate	Low drying efficiency, uneven temperature and humidity, affects product quality
Microwave Drying	High	High (Approx. USD 300–500/tonne)	Significant loss	Hard to control moisture content, high energy consumption, possible surface overheating	Suitable for high moisture content materials	High efficiency, rapid, capable of deep heating, suitable for materials requiring quick drying	High cost, difficult to control moisture, risk of overheating and nutrient loss
Vacuum Drying	High	High (Approx. USD 200–600/tonne)	Minimal	High equipment cost, high energy consumption	Suitable for temperature-sensitive high-value foods and medicinal materials	Maximizes nutrient retention, preserves flavor and quality	High equipment cost, operational and maintenance cost, high energy consumption
Freeze Drying	High	High (Approx. USD 500–1000/tonne)	Minimal	High initial investment, long processing time, low energy efficiency	Suitable for high-end products like fruits, vegetables, and seeds	Best for temperature-sensitive materials, preserves nutrients and sensory quality	High equipment and maintenance costs, long drying cycle, low energy efficiency
Infrared Drying	High	Moderate (Approx. USD 150–300/tonne)	Minimal	Faster drying process, but limited penetration, often used in combination with other technologies	Suitable for high moisture materials, especially grains and fruits	Even heating, fast drying, suitable for materials with higher surface moisture	Limited penetration, not effective for thick or low moisture materials, typically needs to be combined with other technologies
Solar Drying	Medium to High	Low (Approx. USD 10–50/tonne)	Minimal to moderate (depends on drying time and temperature control)	Dependent on solar radiation, weather, and geographic location; some systems include heat storage for night-time operation	Small to medium-scale production, especially in areas with abundant sunlight	Low operating cost, eco-friendly, suitable for remote areas, can preserve nutrients better than conventional drying	Weather-dependent, drying efficiency can fluctuate, requires proper system design for optimal performance, initial setup cost

## Data Availability

No new data were created or analyzed in this study. Data sharing is not applicable to this article.
